# Critical appraisal and concerns regarding a meta-analysis on prothrombin complex concentrate (PCC) for trauma-induced coagulopathy: unveiling methodological nuances and treatment variances

**DOI:** 10.1186/s13054-023-04743-9

**Published:** 2023-11-21

**Authors:** Bruno Caldeira Antônio, Maiara Sulzbach Denardin, Henrique Alexsander Ferreira Neves, Eduardo Messias Hirano Padrao

**Affiliations:** 1 Federal University of Paraná, Curitiba, Brazil; 2 Department of Pulmonary and Critical Care, Massachusetts General Hospital, Harvard Medical School, Boston, MA USA

To the Editor,

We read with interest the paper “Prothrombin complex concentrate (PCC) for treatment of trauma-induced coagulopathy: systematic review and meta-analyses” [1]. We appreciate our colleagues’ efforts in advancing our understanding of PCCs in trauma patient management. However, it is crucial to highlight that the primary goal of a systematic review and meta-analysis is to precisely determine effect sizes, which requires the exclusion of studies that could induce bias in the results. This paper drew our attention due to the apparent absence of strict criteria for including studies in the meta-analysis.

The first study of concern, conducted by Khurrum et al. [2], compared patients in the intervention group treated with four-factor prothrombin complex concentrate (4F-PCC) and whole blood (WB) to those in the control group treated only with WB. While the aim of the article is to assess the impact of 4F-PCC, we note the inconsistency in comparing studies that use WB with others utilizing fresh frozen plasma (FFP) as the control treatment. Recent research suggests that WB displays an improved functional clotting profile compared to the conventional 1:1:1 transfusion ratio of packed red blood cells, fresh frozen plasma, and platelets [3].

The second concerning study was conducted by Schlimp et al. [4]. Unlike the other studies using FFP as the control treatment, this study employed fibrinogen concentrate (FC) instead. FFP has low doses of fibrinogen, posing challenges in maintaining fibrinogen blood concentration with plasma alone during resuscitation. Additionally, while the other studies in the meta-analysis excluded patients on anticoagulation, the authors explicitly state that it was not a criterion of exclusion. Moreover, examining the results, we note that the intervention group (PCC + FC) had 18 deaths out of 63 patients, while the control group (FC) had 7 deaths out of 85 patients (29% vs. 8%, respectively, *p* = 0.0001). However, it is noticeable that the intervention group exhibited higher injury severity score, base deficit, lactate concentration, and a lower pH than the control group, differences that influence mortality rates [4].

It is noteworthy how the articles utilize either 3F-PCC or 4F-PCC for trauma-induced coagulopathy (TIC) treatment. The primary distinction between the two forms of PCC lies in the higher concentration of coagulation factor VII and the inclusion of anticoagulant proteins in 4F-PCC [5]. Despite the similarities in composition, studies show that 4F-PCC is likely more effective than 3F-PCC in reducing the international normalized ratio and the need for blood transfusions [6, 7]. Therefore, a deeper exploration of the differences between these two formulations could help optimize TIC treatments.

Addressing these concerns, we recalculated the odds ratio (OR) for the mortality outcome without Khurrun et al. and Schlimp et al. [2, 4, 8–12]. Additionally, we separated 3F-PCC and 4F-PCC articles in subgroup analysis. Given the expected heterogeneity between the studies, we performed a random-effect meta-analysis using the inverse variance method (Fig. 1) [13]. In our analysis, the use of PCC is associated with reduced mortality (OR 0.68, 95% confidence interval [CI] 0.51–0.90, *I*^2^ 0%), contrasting with the review’s results (OR 0.94, 95% CI 0.60–1.45, *I*^2^ 64%) [1]. In addition, our corresponding heterogeneity was much lower (*I*^2^ 0%) than that presented by Hannadjas and colleagues (*I*^2^ 64%) [1]. The subgroup analysis showed a reduction in mortality rates with 4F-PCC (OR 0.65; 95% CI 0.47–0.89, *I*^2^ 0%), while the 3F-PCC group exhibited no significant impact (OR 0.79; 95% CI 0.45–1.38, *I*^2^ 0%).

**Fig. 1:**
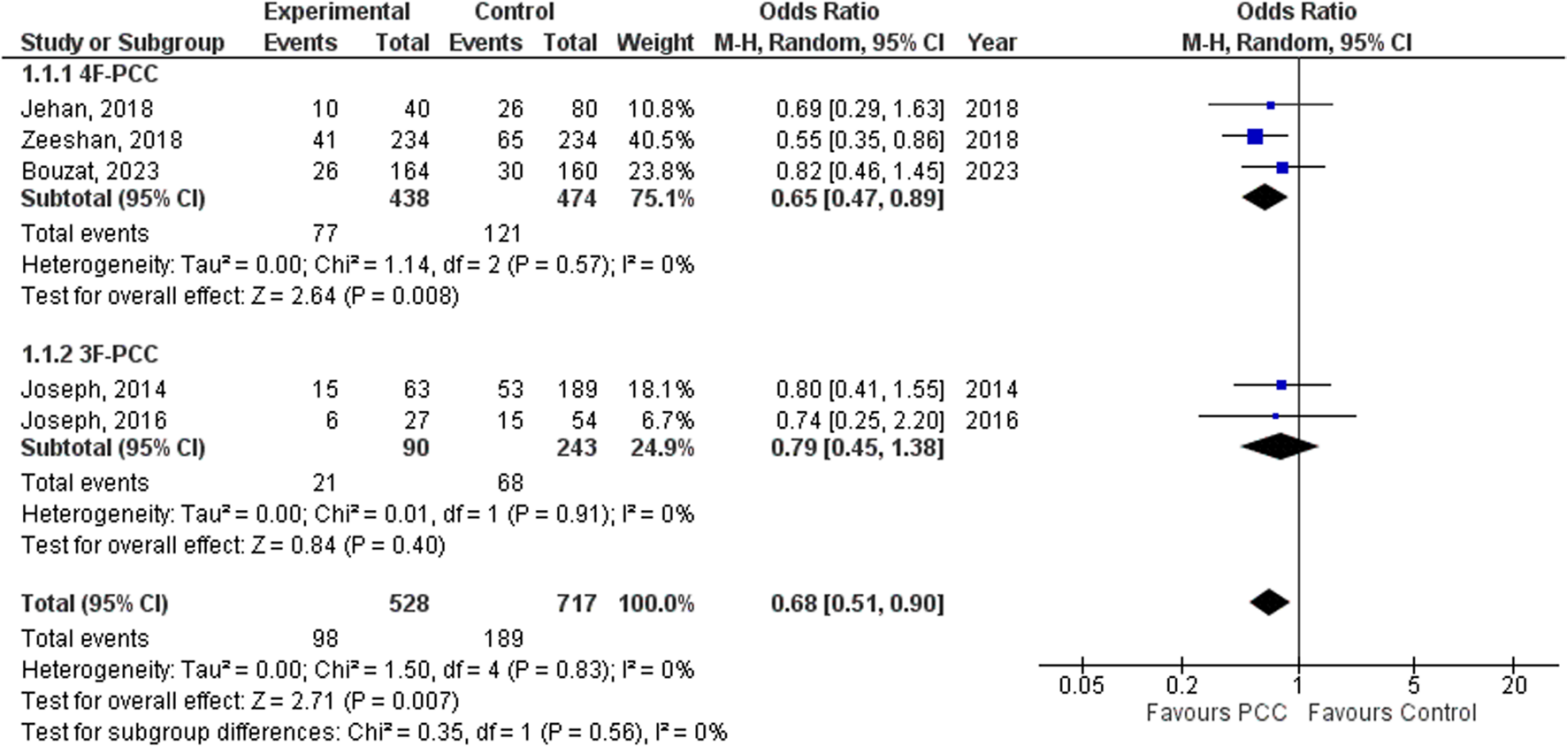
Forest plot comparison of mortality in patients treated with PCC vs control treatment in subgroup analysis

To explore the heterogeneity in the review, we propose Baujat’s graphic method, which detects sources of heterogeneity and assesses their contribution to the overall result [14]. In Fig. 2, each study is represented by a dot on the graph. Notably, Schlimp et al. [4] stand out as the primary contributor to the overall heterogeneity and moderate influence on the overall result. While Khurrum et al. [2] showed some treatment variations, their contribution to overall heterogeneity was not significant.

**Fig. 2:**
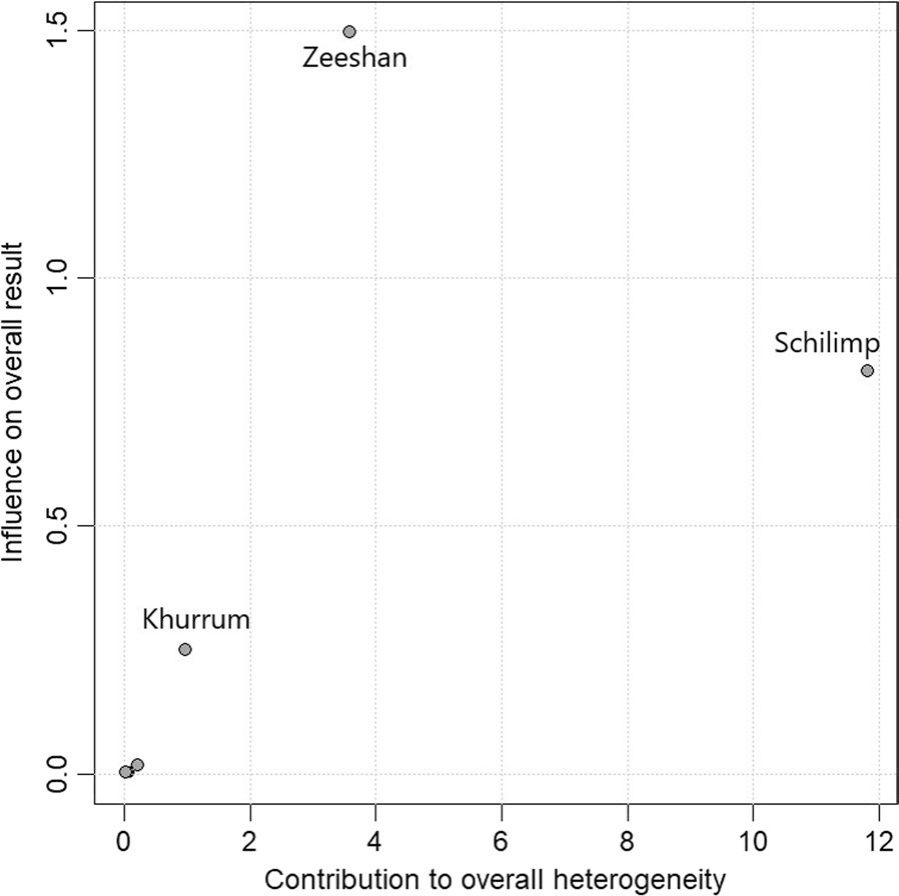
Baujat’s graphic method

To enhance our comprehension of the impact of individual studies on the effect size and heterogeneity, we conducted a leave-one-out meta-analysis, as illustrated in Fig. 3. Upon excluding Schlimp et al. from the meta-analysis, a significant shift in effect size is observed in favoring 4F-PCC, along with a substantial reduction in heterogeneity (the *I*^2^ values decrease from 64 to 0%) [4].

**Fig. 3:**
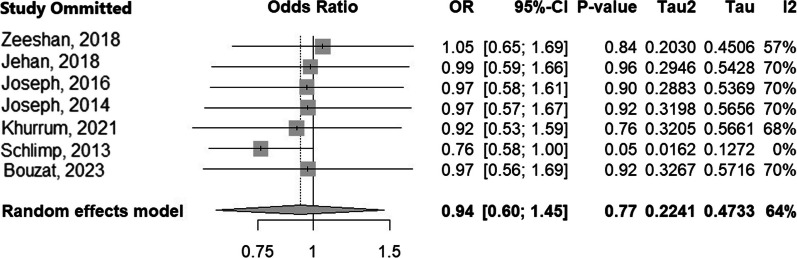
Leave-one-out meta-analysis method

Finally, the inclusion of Schlimp et al. [4] appears inappropriate due to its impact on overall results, heterogeneity, and inadequate comparability with other studies. Meticulous study selection is essential for unbiased and precise inferences. Therefore, our methodological approach has led to conclusions that diverge from those presented by the original author. However, we agree with the authors that drawing a recommendation would be warranted with the publication of additional randomized trials. Moreover, a comprehensive understanding of the distinctions between 4F-PCC and 3F-PCC in TIC treatment requires further studies.

## Data Availability

Not applicable.

## Abbreviations

3F-PCC: Three-factor prothrombin complex concentrate
4F-PCC: Four-factor prothrombin complex concentrate
CI: Confidence interval
FC: Fibrinogen concentrate
FFP: Fresh frozen plasma
OR: Odds ratio
PCC: Prothrombin complex concentrate
TIC: Trauma-induced coagulopathy
WB: Whole blood
